# Evaluating the effectiveness of incentives to improve HIV prevention outcomes for young females in Eswatini: Sitakhela Likusasa impact evaluation protocol and baseline results

**DOI:** 10.1186/s12889-020-09680-8

**Published:** 2020-10-22

**Authors:** Marelize Gorgens, Andrew F. Longosz, Sosthenes Ketende, Muziwethu Nkambule, Tengetile Dlamini, Mbuso Mabuza, Kelvin Sikwibele, Vimbai Tsododo, Mthokozisi Dlamini, Futhie Dennis-Langa, Wendy Heard, Andrea Low, Pandu Harimurti, David Wilson, Khanya Mabuza, Damien de Walque

**Affiliations:** 1 The World Bank Group, 1776 G Street, Washington, DC, 20006 USA; 2 National Emergency Response for HIV/AIDS, Mbabane, Eswatini; 3 IHM Southern Africa, Mbabane, Eswatini; 4 Independent, Mbabane, Eswatini

**Keywords:** CCT, Eswatini, Education, Females, HIV, Adolescence, Cash-incentivised

## Abstract

**Background:**

Eswatini continues to have the highest prevalence of HIV in the world, and one of the highest HIV incidences among adult populations (aged 15–49). This analysis reports on both key elements of study design/protocol and baseline results from an impact evaluation of an intervention incentivizing (i) initiation, enrolment, attendance or completion of some form of education, and (ii) lower risk sexual behaviour.

**Methods:**

The impact evaluation employs a two by two factorial design in which participants are enrolled in either the incentive for education arm (‘education treatment arm’ providing a conditional cash incentive) or the control arm (‘education control arm’). In each of these arms, 50% of participants were randomized to also be eligible for selection – three times a year – to participate in a conditional raffle conditional on testing negative for curable STIs (syphilis and *Trichomonas vaginalis*).

**Results:**

Baseline recruitment and screening occurred in 2016 when a total of 6055 individuals were screened of which 4863 participated in the baseline survey, and 4819 individuals were randomized into one of the study arms. The baseline prevalence of HIV, *Trichomonas vaginalis*, and syphilis among adolescent girls and young women 8.20% (397/4840), 3.31% (150/4533) and 0.17% (8/4830) respectively.

**Conclusions:**

An educational cash incentive and raffle incentive impact evaluation that addresses adolescent girls and young women who are in-education and out-of-education has the potential to reduce HIV risk in adolescent girls and young women in Eswatini.

**Trial registration:**

Name of the registry: Pan African Clinical Trials Registry.

Trial registration number: PACTR201811609257043.

Date of registration: May 11, 2018 ‘Retrospectively registered’.

URL of trial registry record: https://pactr.samrc.ac.za/TrialDisplay.aspx?TrialID=4685

## Background

The highest rates of HIV infection are in southern Africa, with more than 1% of the population per year becoming infected in Botswana, Lesotho and Eswatini [1]. There are 6 countries (Botswana, Lesotho, Namibia, Eswatini, South Africa, and Zimbabwe) that have HIV prevalence of more than 10% of the entire population [1]. In 2015, Eswatini had an estimated 13,910 new HIV infections, 263,040 people living with HIV (PLHIV), and 5890 HIV/AIDS related deaths [2]. Some of the main factors associated with transmission of HIV in Eswatini include: low prevalence of male circumcision; multiple, long-term concurrent sexual relationships; early sexual debut and intergenerational sex; low condom use, especially in long-term sexual partnerships; lack of family and community support; and multiple structural factors [3, 4].

The three main structural factors that influence HIV incidence include income inequality, gender inequality, transactional sex and education [4–7]. Income inequality has been shown to be a major contributor to sexual risk in women in Eswatini [8]. Eswatini is classified as a lower Middle Income country by the World Bank [9]. Yet, Eswatini has one of the highest levels of income inequality in the world: despite it being a lower middle income country, 63% of the population living on less than $2 per day [9]. The poor economic prospects for young women contribute to the increasing prevalence of intergenerational and transactional sex, particularly as young women develop desires and needs consistent with modern expectations of lifestyle. A qualitative study of girls in rural areas of Eswatini found that lack of access to education and employment, and food insecurity often pushed young women into intergenerational partnerships where there was the potential for economic gain [7]. The Ministry of Education of Eswatini provides both Comprehensive Sexual Education and Information and Life Skills education within the schools’ syllabi. However, school fees and other contextual considerations may limit the ability of adolescent girls and young women from attending school. Therefore, overcoming economic barriers will be important for adolescent girls and young women to gain an understanding of reproductive health and sexual transmitted disease prevention. Additionally, gender-based violence is a widespread problem in Eswatini where the United Nations Populations Fund (UNFPA) estimates that 1 in 3 females experienced some form of sexual abuse by the age of 18 years, and 48% of women report to have experienced some form of sexual based violence in their life time [10].

There have been mixed results from recent cash incentive studies for HIV prevention and their impact on HIV and other sexually transmitted infections. Evidence obtained from cash incentive studies show that cash incentives may reduce HIV [11, 12] and STI (sexually transmitted infection) or HSV-2 (herpes simplex virus 2) incidence [11–14] but not all studies demonstrate significant reductions [15]. Additionally, few studies currently focus on out-of-education young women [11] while this group could benefit greatly from cash incentives for HIV prevention as well as more broadly to improve their wellbeing. One important limitation across cash transfer studies are that several of these studies only include girls already enrolled in school and focus on urban areas [13, 15]. Additionally, raffles have also been proposed and evaluated as a financial incentive to reduce STIs [12]. However, raffles have not been evaluated in addition to education incentives to determine if there is a compound effect of these cash incentives.

**Fig. 1:**
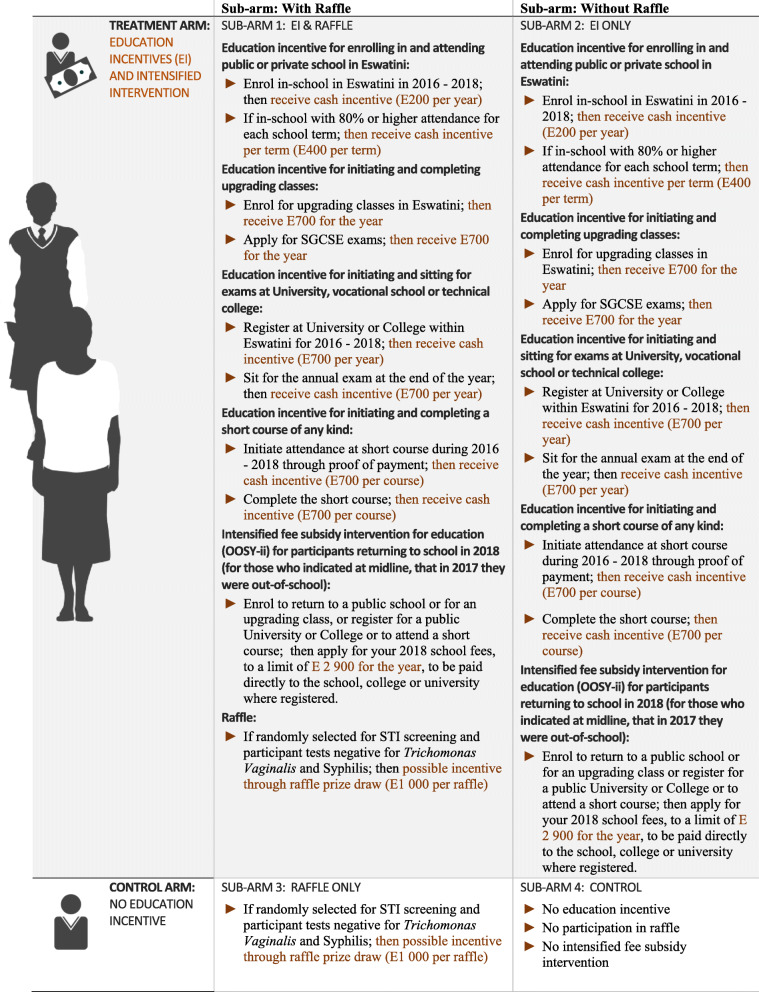
Description of impact evaluation and sub arms, with interventions assigned to each group. Image generated using Adobe Illustrator and Photoshop

The Sitakhela Likusasa Impact Evaluation rationale and study design combines the study strengths of these 5 studies: it covers a large age range, includes urban and rural settings, and multiple intervention arms. The four intervention arms for the study follow a 2 × 2 factorial design and are presented in Fig. 1.

Given the number of countries with cash incentive programmes in place, undertaking studies to assess the impact of cash incentives on HIV incidence reduction for countries with generalized HIV epidemics is important [16]. This impact evaluation will provide complementary data to the studies detailed above, including the most vulnerable girls (education dropouts) and those with poor education outcomes prior to incentives. Our hypotheses are 1) that cash incentives conditional on education attendance will allow girls to continue or return to places of education and reduce high risk sexual behaviour, and 2) that raffle based incentives to girls conditional on a negative curable STI status will reduce risky behaviour, by providing incentives to minimise risk. Analysis in this report aims to describe key aspects of the overall protocol design of the study and report baseline results on key risk behaviours and sexual reproductive health outcomes.

## Methods

### Study design and study population

This study was designed to assess the impact of incentives for education attendance/enrolment and remaining STI negative on HIV incidence among HIV negative adolescent girls and young women (AGYW) aged 15–22 at baseline. AGYW were eligible for education enrolment at the primary, secondary, or tertiary, provided informed consent (or had a legal guardian to do so), consented to HIV testing, consented to a urine collection for trichomonas confirmation testing and consented to blood work and regular vaginal swabs for STI testing. The study included AGYW who dropped out of education but were still eligible to return, in order to assess whether incentives could help overcome other factors driving education dropouts. Because of the high HIV-1 incidence amongst AGYW, this evaluation was designed to assess the impact and cost-effectiveness of two different incentives – (a) conditional cash incentives that were conditional on education attendance among AGYW aged 15–22, and (b) raffle incentives for which raffle enrolment was conditional on being negative for curable STIs (syphilis and *Trichomonas vaginalis*). The primary outcome was HIV-1 incidence at 3 years after study enrolment. Secondary outcomes included *Trichomonas vaginalis* and syphilis prevalence at endline, and changes in reported sexual behaviours between baseline and endline. Additional outcomes included education enrolment and attendance and graduation rates, cost-effectiveness of cash incentives in terms of HIV prevention, cash incentive cost-effectiveness on educational outcomes, and the effects of a raffle incentive on enhancing a cash incentive intervention.

In order to investigate the impact of cash incentives on HIV incidence the study used a 2 X 2 factorial design to allow for a more efficient use of study resources by reducing the required sample size. At a cluster level, 50% of the participants in the treatment clusters will receive a cash incentive for education, and at the individual level within each treatment arm, 50% of the participants will be enrolled in a raffle conditional on testing negative for curable STIs. The aim was to enrol, from November 2015 to April 2016, 4300 girls in 166 randomly selected census enumeration areas (EAs). Three-stage sampling and randomization took place: first, EAs were filtered, randomly selected and randomly allocated to either education treatment or education control arms (50% in the treatment arm and 50% in the control arm); second, households with potential participants were randomly identified and all eligible participants within those households were invited to enrol in the study; and third, all enrolled participants were randomly allocated to either the raffle treatment or raffle control arms. For the EA-level sampling, we first selected, from the universe of all EAs in Eswatini, a subset of EAs that met specific conditions: EAs with both a population density of at least 100 and with a female population of at least 200 (based on results from the 2007) census. Furthermore, EAs that were within or adjacent to the EAs where the Orphans and Vulnerable Children (OVC) impact evaluation (a study evaluating unconditional cash transfers for orphans and vulnerable children) was taking place, were excluded. The second stage of EA sampling involved stratified random sampling of 400 EAs (20% urban EAs and 80% rural EAs). Then 166 EAs were randomly sampled from the 400 EAs using a random number generator and allocated to education treatment or education control arms. Because EAs were oversampled (i.e. more than the 166 EAs needed) treatment and control EAs that were adjacent to each other could be and were replaced (through random selection) with another of the 400 already-randomly selected EAs (an urban EA would replace another urban EA, and so on). This resulted in treatment and control EAs that were neither adjacent to the OVC study EAs, nor immediately adjacent to each other.

**Fig. 2:**
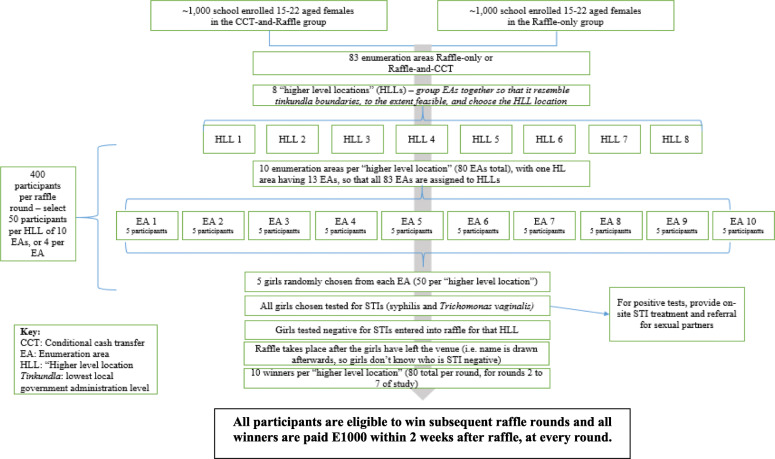
Raffle Intervention Procedure during rounds 2 to 7

For the participant-level random selection, the household listing from the 2007 census was used. Where the household listing from the census was not available, a new household listing was done. In each selected EA, ‘seed’ households were randomly selected using geospatial sampling. This sampling process involved overlaying the selected EA map over Google Earth, and randomly dropping a location pin in that EA using geospatial analysis software. The household closest to the pins was sampled first, and then every 4th household after that. The aim was to keep sampling households (and potential participants) until, within each EA, there would be 13 AGYW who were attending education at the time of enrolment into the study and 13 AGYW who were not in education. Achieving this target of 26 participants per EA, was difficult. Sampling amongst the initial set of 166 EAs did not yield the targeted 4300 AGYW. The two main reasons for having to use additional EAs were 1) difficulty of enrolling out of education AGYW, and 2) difficulty recruiting urban AGYW. Therefore, additional EAs were randomly selected from the 400 EAs initially sampled during the first stage of EA sampling. An additional 127 EAs were added to ensure there was a sufficient number of urban and out of education AGYW enrolled in the study, using the same conditions as for the first 166 EAs and ensuring that the 80/20 rural/urban split was maintained and that treatment and control EAs would not be adjacent to each other. Once participants’ HIV status was determined and they opted to enrol into the study, they were randomly allocated to a raffle treatment arm or raffle control arm, creating 4 sub-arms: education only sub-arm, education-and-raffle sub-arm, raffle only sub-arm and no intervention sub arm (see Fig. 2).

### The education cash incentive intervention

To better understand what interventions might be most useful in Eswatini, we reviewed relevant literature and discussed feasibility and policy relevance of different incentives with colleagues at National Emergency Response for HIV/AIDS (NERCHA). Based on our discussions we found there to be a larger effect of cash incentives on education outcomes with monitoring and enforcement of the conditionality than simply providing unconditional cash transfers [16].

The initial design of the intervention was that cash incentives, conditional on either of the following criteria, would be provided throughout the intervention period:

- E200 (~ 13.40 USD) for enrolling in primary or secondary education in a given education year
- E400 (~ 26.80 USD) per education term for attending more than 80% of their classes each education term over the study implementation period
- For a randomly selected sub-sample of the participants: E1000 (~ 67.00 USD) for the winners of a raffle in which those who tested negative for two curable STIs (*Trichomonas vaginalis* and syphilis) were entered

These amounts were decided in consultation with the government of Eswatini, balancing the need to propose amounts that would provide strong enough incentives while maintaining a transfer size reasonable within the Eswatini context and that could potentially be scaled-up by the government.

After the first year of implementation, we received feedback from the Ministry of Education’s regional guidance officers that there were several AGYW who attended upgrading classes and not traditional education. Older AGYW within our study population also attended upgrading classes, or short courses, or university or other vocational training and they were being excluded from receiving a cash incentive for attending some form of education. Based on this information we determined that from year 2 of implementation onwards, attending upgrading classes, short courses, university or vocational training should all be considered as being eligible for cash incentives. In addition, the midline data collection effort revealed that one of the main factors of AGYW not attending education or enrolling in education were lack of registration fees. Therefore, we also designed an added incentive to pay the education registration fees of all AGYW in the education treatment arm who were out of education as of midline data collection. Consequently, after the first year of study implementation (2016), a protocol amendment was approved whereby it was clarified how incentives for other forms of education than formal education would be paid to participants in the education treatment arm, as follows:

- *Education incentive for initiating and completing upgrading classes:*
  - Enrol for upgrading classes in Eswatini; then receive E700 (~ 46.81 USD)
  - Apply for O level exams; then receive E700 (~ 46.81 USD)
- *Education incentive for initiating and sitting for exams at University, vocational school or technical college:*
  - Register at University or College within Eswatini for 2016 and/or 2017; then receive cash incentive of E700 (~ 46.81 USD) per year
  - Sit for the annual exam at the end of the year; then receive cash incentive of E700 (~ 46.81 USD) per year
- *Education incentive for initiating and completing a short course of any kind:*
  - Initiate attendance at short course during 2016 and/or 2017 through proof of payment; then receive a cash incentive of E700 (~ 46.81 USD) per course
  - Complete the short course; then receive a cash incentive of E700 (~ 46.81 USD) per course
- *Education incentive for participants returning to education in 2018 who were out-of-education in 2017*
  - Enrol to return to a public education or for an upgrading class or register for a public University or College or to attend a short course; then apply for your 2018 education fees, to a limit of E2,900 (~ 193.94 USD) for the year, to be paid directly to the education, college or university where registered.

Payments were made using MTN Mobile Money for participants 18 years or older (being 18 years of age is a requirement in Eswatini for registering for MTN Mobile Money, a cell phone-based money transfer service). Those younger than 18 years could elect to either receive their incentives through the Swaziland Post and Telecommunications office, or for it to be given to the parent/guardian with the expectation that the payment would be shared with the participant. In all cases, actual payments to participants were verified telephonically (to verify that the participant received the funds).

### The STI raffle intervention

Out of the study participants who were randomized to either the ‘education and raffle incentive’ sub-arm or the ‘raffle incentive’ sub-arm (Fig. 2), 400 were selected every raffle round to participate in STI testing (syphilis and *Trichomonas vaginalis*). Participants were not excluded or reallocated if more than one individual per household was chosen. The possibility to select more than one individual per household was well understood and did not create problems during field work. OSOM *Trichomonas vaginalis* rapid test and Alere Determine syphilis rapid test were used to determine STI status. Positive results were provided through post-test counselling by a trained nurse. Upon being STI negative, these randomly selected participants were then eligible to win one of 80 prizes of E1000 each. For anyone with a positive rapid test, the counsellor obtained a detailed history to determine the duration of infection, including a history of previous syphilis, known contact with someone with primary or secondary syphilis, and typical signs or symptoms of syphilis in the past 12 months. Participants who tested positive for either syphilis or *Trichomonas vaginalis* were given treatment and retested after 2 weeks. If the AGYW tests negative after 2 weeks of treatment, she was eligible for selection for the next raffle round.

### HIV and STI counselling, testing, treatment and referral

HIV testing consisted of the Alere Determine HIV-1/2 rapid test kit and those who were HIV positive by the Determine HIV-1/2 rapid test and/or test inconclusive received the Uni-Gold rapid test for confirmation. HIV tests were administered at study testing sites by nurses and healthcare workers certified in HIV testing and counselling. Any individual testing positive for HIV was referred to a healthcare facility for treatment. All study participants were counselled before testing by a HIV Services Testing Counsellor. Participants were tested at baseline, midline and endline each test was approximately 1 year apart. At each interaction with the study team (whether for intervention implementation or data collection), study participants were screened for gender-based violence (GBV) using a screening tool developed in partnership with the Eswatini Action Group Against Abuse (SWAGAA). Throughout the study, suspected GBV cases were referred to SWAGAA for follow-up and support as part of their routine counselling and support mechanisms.

### Data collection plan and analyses

Data were generated from questionnaire data, education enrolment and attendance data, raffle results and laboratory results. In terms of data collection: (a) Data on education attendance were collected from routine sources using the Ministry of Education, education attendance registers, (b) In addition, spot checks were done to confirm education attendance, (c) Costing data relating to the implementation of each incentive were collected throughout the study to allow cost-effectiveness estimation as a function of the estimated HIV infections averted, and (d) Data collection for sexual behaviour was collected at baseline, midline and endline questionnaires by field data collectors. Data captured during the evaluation were uploaded using personal digital assistants (PDAs) in the field, to ensure consistent data capture across districts. Questionnaire data, education enrolment and attendance data, raffle results and laboratory results were entered into SurveyToGo and processed with a bespoke data management system developed in SQL. After validation of duplicate files, the data were then exported to a statistical package (STATA) for further cleaning and analysis.

### Analysis included in this report

Baseline data was collected at the time of participant enrolment in 2016. The objective of the baseline data collection and analysis was twofold: describe the sociodemographic characteristics of the study population and identify variables affecting school enrolment and HIV and STI status because they could act as confounding characteristics which would need to be controlled for in the impact evaluation. The purpose of the baseline data analysis was to ascertain the following information about the study population: describe demographic characteristics; report study level education enrolment, and investigate characteristics and factors associated with education enrolment at baseline; report study level HIV and STI prevalence, and investigate relationships between background characteristics of participants, and HIV and STI status at baseline. Table 1 provides key baseline socio-demographic and descriptive evidence about the sample composition.

**Table 1:** Characteristics of adolescent girls and young women at baseline

Characteristics	All Participants (***N*** = 4863)	Urban (U) (***N*** = 895)	Rural (R) (***N*** = 3968)	U = R ***p-***value	^**a**^Enrolled-in-Education (EIE) (***N*** = 2337)	Not ^**a**^Enrolled in-Education (NIE) (***N*** = 2526)	EIE = NIE ***p-***value
**Age in years (mean (SD))**	18.22 (2.24)	18.16 (2.28)	18.24 (2.24)		16.94 (1.90)	19.40 (1.86)	
15 years (n (%))	779 (16.0%)	143 (16.0%)	636 (16.0%)	**0.16**	693 (89.1%)	86 (11.0%)	**< 0.01**
16 years (n (%))	594 (12.2%)	134 (15.0%)	460 (11.6%)		473 (79.6%)	121 (20.4%)	
17 years (n (%))	628 (12.9%)	115 (12.9%)	513 (12.9%)		405 (64.5%)	223 (35.5%)	
18 years (n (%))	612 (12.6%)	104 (11.6%)	508 (12.8%)		281 (45.9%)	331 (54.1%)	
19 years (n (%))	634 (13.0%)	104 (11.6%)	530 (13.4%)		194 (30.6%)	440 (69.4%)	
20 years (n (%))	636 (13.1%)	108 (12.1%)	528 (13.3%)		143 (22.5%)	493 (77.5%)	
21 years (n (%))	592 (12.2%)	112 (12.5%)	480 (12.1%)		98 (16.6%)	494 (83.5%)	
22 years (n (%))	388 (8.0%)	75 (8.4%)	313 (7.9%)		50 (12.9%)	338 (87.1%)	
**School history**							
Highest grade attained							
Grade 1–7 (n (%))	1156 (23.8%)	182 (20.3%)	974 (24.6%)	**< 0.01**	550 (47.6%)	606 (52.4%)	**< 0.01**
Form 1–6 (n (%))	3581 (73.6%)	668 (74.6%)	2913 (73.4%)		1700 (47.5%)	1881 (52.5%)	
Year 1–5 (n (%))	126 (2.6%)	45 (5.0%)	81 (2.0%)		87 (69.1%)	39 (31.0%)	
^b^Time travelled to school > 30 min (n(%))	832 (36.4%)	144 (28.5%)	688 (38.6%)		–	–	
**Sexual behaviours**							
Never had sexual intercourse (n (%))	2301 (47.3%)	477 (53.3%)	1824 (46.0%)	**< 0.01**	1785 (77.6%)	516 (22.4%)	**< 0.01**
^c^Sexual partner age mixing (n (%))	428 (20.3%)	77 (22.5%)	351 (19.8%)	0.27	67 (15.6%)	361 (84.4%)	**0.01**
Sexual intercourse multiple partners in last 12 months (n (%))	25 (1.2%)	7 (2.0%)	18 (1.0%)	0.11	7 (28.0%)	18 (72.0%)	0.32
Condom used with last partner (n (%))	1377 (65.1%)	242 (70.6%)	1135 (64.1%)	**0.02**	334 (24.3%)	1043 (75.7%)	**< 0.01**
Received money or gift in exchange for sexual intercourse (n (%))	586 (27.7%)	88 (25.7%)	498 (28.1%)	0.35	99 (16.9%)	487 (83.1%)	**0.03**
Ever given birth (n (%))	1331 (52.0%)	159 (38.0%)	1172 (54.7%)	**< 0.01**	83 (6.2%)	1248 (93.8%)	**< 0.01**
First pregnancy before the age of 17 (n (%))	505 (37.9%)	63 (39.6%)	442 (37.7%)	0.64	34 (6.7%)	471 (93.3%)	0.56
Last sexual partner circumcised (n (%))	782 (42.3%)	152 (52.6%)	630 (40.4%)	**< 0.01**	163 (20.8%)	619 (79.2%)	**< 0.01**
**HIV and Sexual Practices Knowledge**							
Heard of AIDS (n (%))	4702 (96.7%)	869 (97.1%)	3833 (96.6%)	0.45	2245 (47.8%)	2457 (52.3%)	**0.02**
Knows that a healthy looking person can have HIV (n (%))	4092 (84.2%)	757 (84.6%)	3335 (84.1%)	0.69	1915 (46.8%)	2177 (53.2%)	**< 0.01**
^c^Heard of age mixing (n (%))	3278 (67.6%)	573 (64.7%)	2705 (68.3%)	**0.04**	1503 (45.9%)	1775 (54.2%)	**< 0.01**
Heard of transactional sex (n (%))	3429 (71.0%)	603 (68.1%)	2826 (71.6%)	**0.04**	1596 (46.5%)	1833 (53.5%)	**< 0.01**
**Household structure**							
Mother alive (n (%))	4057 (84.0%)	759 (85.7%)	3298 (83.6%)	0.13	2002 (49.4%)	2055 (50.7%)	**< 0.01**
Father alive (n (%))	3189 (67.0%)	608 (69.1%)	2581 (66.5%)	0.15	1622 (50.9%)	1567 (49.1%)	**< 0.01**
Female-headed household (n (%))	1592 (32.7%)	336 (37.5%)	1256 (31.7%)	**< 0.01**	739 (46.4%)	853 (53.6%)	0.11
Number of siblings (mean (SD))	1.46 (1.2)	1.29 (1.1)	1.49 (1.2)	**< 0.01**	1.54 (1.2)	1.38 (1.2)	**< 0.01**
Currently married or in union (n (%))	115 (2.4%)	11 (1.2%)	104 (2.6%)	**0.01**	0 (0.0%)	115 (100.0%)	**< 0.01**

^a^Enrolled in education: This variable corresponds to if an individual said she was enrolled in education at the time of the interview

^b^Time travelled to school: How long does it take the AGYW to travel from their house to school regardless of the time of day in minutes. Not answered by those not enrolled in education

^c^Age mixing defined as having sexual intercourse with an individual 10 years or older than one’s self in the last 12 months

*P*-value are generated to show if there was a significant difference in Reponses between urban versus rural, and comparing enrolled in school and not enrolled in school; significant observation (*p*-value < 0.05) are **bolded**

Responses of “Don’t know,” “Refuse to answer,” and “Not Applicable” are not included in this table. Missing values due to skip patterns are also not included in this table

Column totals and percentages were calculated for locality, and row totals and percentages were calculated for in-school versus out-of-school

In order to test for a possible relationship between background characteristics of participants, and HIV and STIs prevalence a chi-square test was used to test the significance of possible association between those characteristics, and HIV and STIs infection. For inadequate data where the expected data in the contingency table cell are < 5, the Fisher’s exact test will be performed as an alternative test. A cross-sectional analysis was also performed for baseline data, examining determinants of HIV infection using logistic regression, with random effects to adjust for intra-cluster correlation. Independent variables of interest included baseline education enrolment, socio-economic status, geographic location, age, age at sexual debut, history of intergenerational sex, transactional sex, sexual partner characteristics, circumcision status of partner and prevalence of other STIs.

The collection and testing of vaginal swabs in all participants at baseline provided cross-sectional data on the prevalence of *Trichomonas vaginalis*, which had not been surveyed in Eswatini since 2005. Serological testing for syphilis also provided baseline data on prevalence. The random selection of EAs and girls ensured that the sample was relatively representative of young women and girls across Eswatini, although the sample size was not powered to provide estimates representative of the entire population. The prevalence of each STI was calculated as a proportion of positive from the total population sampled.

## Results

**Fig. 3:**
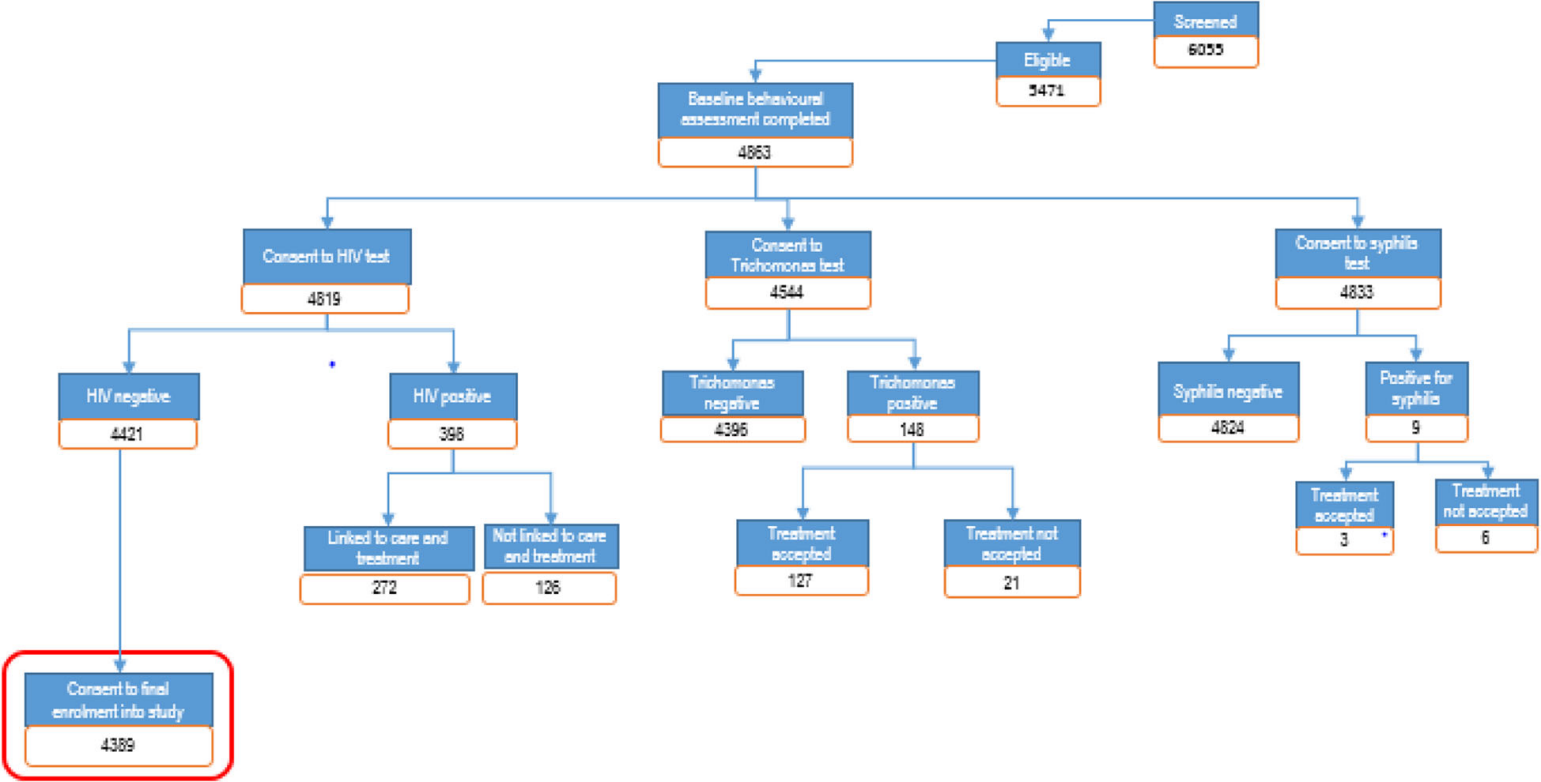
Baseline enrolment cascade

At baseline 6055 individuals aged 15 to 22 years were screened of which 4863 participated in the baseline survey, 4421 were HIV negative and 4389 chose to enrol into the study. As designed, 50.4% of those enrolled in the study were enrolled-in-education at the start of the study with ~ 50% in education and ~ 50% out-of-education within each of the study groups (Table 1). As designed across the study groups ~ 80% of the individuals were from rural enumeration areas (EAs) and ~ 20% were from urban EAs (Table 1). Figure 3 shows the cascade from screening to enrolment for the impact evaluation. The mean age of the sample was 18.22 years (standard deviation [SD]: ± 2.24). There were multiple significant differences (*p*-value < 0.05) when comparing urban versus rural individuals which included highest grade attained (fewer *n* = 182 (20%) AGYW attaining grades 1–7 for urban and more *n* = 974 (25%) AGYW attaining higher grades for urban [*p <* 0.01]), never having sexual intercourse (fewer urban *n* = 477 (53%) than rural *n* = 1824 (46%) never having intercourse [*p* < 0.01]), condom use with a partner (more urban *n* = 242 (71%) AGYW report using a condom than rural *n* = 1135 (64%) AGYW [*p* = 0.02]), ever giving birth (more rural *n* = 159 (38%) than urban *n* = 1172 (55%) AGYW [*p* < 0.01]), last sexual partner circumcised (more urban *n* = 152 (53%) AGYW than rural *n* = 630 (40%) [*p <* 0.01]), heard of age mixing or transactional sex (both higher for rural *n* = 573 (65%) than urban *n* = 2705 (68%) [*p* = 0.04]), and differences in household structure including female-headed households, number of siblings, and currently married or in a union [*p <* 0.01] (Table 1). Additionally, there were significant differences (p-value < 0.05) when comparing AGYW enrolled in-education at the time of baseline collection and not enrolled-in-education at the time of baseline collection. These included age (older individuals being not enrolled in-education [*p <* 0.01]), school history (not enrolled-in-education individuals attained lower grades [*p <* 0.01]), sexual behaviours (not enrolled-in-education more likely of having had sexual intercourse [*p <* 0.01], experienced sexual partner age mixing [*p* = 0.01], used a condom with last partner [*p <* 0.01], received money or gift in exchange for sexual intercourse [*p* = 0.03], had given birth [*p <* 0.01], sexual knowledge (not enrolled-in-education AGYW heard of HIV and had a higher level of knowledge of sexual practices than in-education AGYW) [*p <* 0.01], and household structure differences [*p <* 0.01] (Table 1).

Over half (52.7%) of the AGYW surveyed at baseline had their sexual debut. Additionally, 20.3% of AGYW who had sexual intercourse in the past 12 months, experienced age mixing (sexual intercourse in the last 12 months with an individual more than 10 years older than themselves), and 27.7% indicated that they had sexual intercourse in the exchange of money or gifts (Table 1). 32.7% of households were headed by females (Table 1). Very few (2.4%) AGYW were married or in a union at baseline (Table 1).

The baseline prevalence of HIV, *Trichomonas vaginalis*, and syphilis among AGYW were 8.20% (397/4840), 3.31% (150/4533) and 0.17% (8/4830) respectively (Table 2). The baseline prevalence of HIV among those enrolled-in-education was 3.7% (86/2326) compared to 12.37% (311/2514) among those not enrolled-in-education (Table 2). The baseline prevalence of *Trichomonas vaginalis* among those enrolled-in-education was 1.25% (27/2153) compared to 5.17% (123/2380) not enrolled-in-education. The baseline prevalence of syphilis among those enrolled-in-education was 0.09% (2/2323) compared to 0.24% (6/2507) not enrolled-in-education.

**Table 2:** Prevalence of HIV, syphilis, and *Trichomonas vaginalis* among adolescent girls and young women at baseline

Laboratory-confirmed STI	Number (n)	Prevalence % (n/N)	In-education at Baseline	Out-of-education at Baseline	Rural	Urban
HIV	397	8.20 (397/4840)^a^	3.70 (86/2326)	12.37 (311/2514)	7.92 (313/3950)	9.44 (84/890)
Both HIV and Syphilis	4	0.08 (4/4827)	0.04 (1/2322)	0.12 (3/2505)	0.08 (3/3938)	0.11 (1/889)
Both HIV and *Trichomonas vaginalis*	50	1.10 (50/4531)	0.23 (5/2153)	1.89 (45/2378)	0.91 (34/3727)	1.99 (16/804)
*Trichomonas vaginalis*	150	3.31 (150/4533)	1.25 (27/2153)	5.17 (123/2380)	3.41 (127/3729)	2.86 (23/804)
Syphilis	8	0.17 (8/4830)	0.09 (2/2323)	0.24 (6/2507)	0.15 (6/3941)	0.23 (2/889)
Both Syphilis and *Trichomonas vaginalis*	3	0.07 (3/4532)	0.05 (1/2152)	0.08 (2/380)	0.03 (1/3728)	0.25 (2/804)

^a^23 individuals without HIV test results; 330 individuals without *Trichomonas vaginalis* test results; 33 individuals without Syphilis test results

Factors in Table 3 that were significantly associated with increased risk of HIV infection at baseline were older age, not being enrolled in education. It should be noted that sexual partner age mixing was an extremely strong predictor of HIV with only 0.1% (2/1685) of AGYW who did not engage in age mixing testing HIV positive while 65.5% (275/420) of AGYW who did engage in age mixing tested HIV positive.

**Table 3:** Factors associated with HIV infection in adolescent girls and young women at baseline

Factors	OR (95% CI)	***p-***value
^**a**^**Age in years**		
19–22 years	3.24 (2.60–4.04)	**< 0.01**
**Locality**		
Rural	0.82 (0.64–1.05)	0.12
**School history**		
^b^Enrolled in education	0.30 (0.24–0.37)	**< 0.01**
^c^Highest grade attained		
Grade 1–7	1	
Form 1–6	0.56 (0.45–0.70)	**< 0.01**
Year 1–5	0.61 (0.31–1.18)	0.14
> 30 min to travel to school	1.02 (0.67–1.56)	0.92
**Sexual behaviours**		
Had sexual intercourse	4.18 (3.26–5.36)	**< 0.01**
^c^Sexual partner age mixing	> 100.00 (346.5–3459.4)	**< 0.01**
Sexual intercourse with multiple partners in last 12 months	2.04 (0.81–5.15)	0.13
Condom used with last sexual partner	0.73 (0.57–0.94)	**0.02**
Received money of gift in exchange for sexual intercourse	1.08 (0.82–1.42)	0.60
Ever given birth	1.45 (1.15–1.83)	**< 0.01**
< 17 years old for first pregnancy	1.31 (0.96–1.77)	0.09
Last sexual partner circumcised	0.98 (0.76–1.28)	0.91
**HIV and Sexual Practices Knowledge**		
Heard of AIDS	1.08 (0.61–1.92)	0.80
Does not know that a healthy looking person can have HIV	0.97 (0.74–1.28)	0.84
Heard of age mixing	1.08 (0.87–1.34)	0.49
Head of transactional sex	1.33 (1.05–1.68)	**0.02**
**Syphilis and** ***Trichomonas vaginalis*** **status**		
Has syphilis	10.84 (6.21–18.92)	**< 0.01**
Has *Trichomonas vaginalis*	1.82 (1.59–2.09)	**< 0.01**
**Household structure**		
Mother deceased	1.85 (1.46–2.34)	**< 0.01**
Father deceased	1.56 (0.27–1.92)	**< 0.01**
Female-headed household	1.09 (0.88–1.34)	0.41
Number of siblings		
No siblings	1	
1 sibling	0.56 (0.42–0.74)	**< 0.01**
2 siblings	0.63 (0.47–0.84)	**< 0.01**
> 2 siblings	0.67 (0.52–0.86)	**< 0.01**
Currently married or in union	3.40 (2.18–5.30)	**< 0.01**

*OR* odds ratio

*CI* confidence interval

Significant observation (*p*-value < 0.05) are **bolded**

^b^Enrolled in education: This variable corresponds to if an individual said she was enrolled in education at the time of the interview

^c^Age mixing defined as having sexual intercourse with an individual 10 years or older than one’s self in the last 12 months

Responses of “Don’t know,” “Refuse to answer,” and “Not Applicable” are not included in this table. Missing values due to skip patterns are also not included in this table

Factors in Table 3 that were significantly associated with decreased risk of HIV infection at baseline were being enrolled-in-education, higher grade of attainment, condom use with last partner, not having a deceased parent and having siblings.

## Discussion

At baseline, the impact evaluation was able to meet the evaluation protocol objectives. Participants consisted of 50% in education and 50% out-of-education AGYW at baseline, 80% rural and 20% urban and there was balance obtained across study sub-arms. Additionally, all ethical considerations were maintained including informed consent and HIV/STI counselling. The impact evaluation was able to obtain good internal validity by successfully enrolling the required number of participants into the study and was able to achieve balance amongst the study arms and sub-arms. The impact evaluation was also able to provide good external validity through its in-depth collection of demographic information, HIV and STI prevalence. Beyond meeting protocol and design objectives the baseline survey provided insights into HIV risk factors for AGYW in Eswatini.

Comparing previous country surveys to the baseline survey was challenging because of the difference in age populations being surveyed. The Multiple Indicator Cluster Survey (MICS) [16] focused on those aged 15–49 years of age rather than stratifying by age group. Comparable findings were found when comparing the baseline survey to the Eswatini HIV Estimates and Projections Report 2015 [2], even though this report consists of modelled estimates and not HIV prevalence data. The Eswatini HIV Estimates and Projections Report estimated HIV prevalence to be 6 and 8% among females aged 15–19 and 20–24 years respectively, where the impact evaluation baseline survey found that 8.2% of 15–22 year old females surveyed tested positive for HIV which is in line with previous study estimates. This gives confidence in the sample and allows for the potential impact to be compared against other programs.

There were some differences and similarities when comparing the structure of Sitakhela Likusasa and other financial incentive studies focused on HIV prevention. Baird et al. in Malawi also included out-of- education girls [11] which is a primary focus of Sitakhela Likusasa because out-of-education girls are more likely to be sexually active and have older sexual partners [4, 7]. Additionally, Eswatini has a much lower secondary school attendance that South Africa and other Southern African countries [17]. Both CAPRISA 007 and HPTN 068 financial incentive studies did not recruit out-of-education girls [13, 15]. Both CAPRISA 007 and Baird et al. Malawi studies used cluster designs and Sitakhela Likusasa followed this practice [11, 13]. When comparing the financial incentives provided in CAPRISA 007 versus Sitakhela Likusasa, the total possible amount of incentives was lower in Sitkhela Likusasa (E1400 in Sitakhela Likusasa vs R1750 in CAPRISA per year) [13], but there was a wider range of opportunities to earn education incentives in Sitakhela Likusasa: e.g., primary/secondary education attendance/enrolment, exam sitting, completion of short-courses, etc.

In a more detailed comparison of baseline results CAPRISA enrolled 2949 individuals, 52.8% were females, mean age was 16.8 years, mean age at sexual debut was ~ 15 years, 31.1% were sexually active and HIV prevalence was 4.2% [13]. The Sitakhela Likusasa has more individuals enrolled, a slightly older study population, were older at first sexual debut, has more are sexually active participants and a higher HIV prevalence at baseline.

HPTN 068 enrolled a total of 2533 participants with the median age of 15 years, 3.2% tested HIV positive, 8.9% were ever pregnant, 26.6% ever had sexual intercourse, 6.6% had sexual intercourse before the age of 15 years, 19.7% had a sexual partner over 5 years older, 14.2% engaged in transactional sexual intercourse, 19.2% had concurrent sexual relationships, 9.8% reported sexual abuse, and 6.6% of those enrolled in school missed more than 5 days of school [15]. At baseline, ever being pregnant, ever having sexual intercourse, early age debut, sexual intercourse with a sexual partner over 5 years older and engaging in transaction sexual intercourse were all significantly (*p*-value < 0.05) associated with HIV infection when holding all other factors constant [15]. Sitakhela Likusasa has an older participant population, more AGYW testing positive for HIV, a similar number were ever pregnant, fewer individuals had sexual intercourse with older partners, fewer individuals had concurrent sexual partnerships, more reported sexual abuse, and more individuals missed more days of school. Similar factors were significantly associated (*p-*value < 0.05) with HIV infection including ever having sexual intercourse and sexual intercourse with an older sexual partner.

At baseline RESPECT enrolled 2399 individuals where 50.2% were females and the mean age was ~ 27.5 years. 10.4% of those enrolled achieved an education level above primary school [14]. Most of the individuals enrolled were married (74.7%). 12.4% of individuals at baseline tested positive for trichomonas, 1.7% tested positive for syphilis and 3.5% tested positive for HIV [14]. In comparison, in Sitakhela Likusasa more individuals were enrolled in school, all are females, the mean age was 18.2 years, more (~ 76%) had education levels above primary school but only 4.6% were married or in a union because the surveyed population was on average ~ 10 years younger.

The strengths of the Sitakhela Likusasa Impact Evaluation design are: a 2X2 factorial design that combines a cash incentive for education enrolment and attendance, and a raffle incentive conditional on remaining STI free; concentration on rural populations (80% allocation) compared to urban populations (20% allocation); enrolling 50% of adolescent girls and young women (AGYW) currently enrolled in education, and 50% of AGYW not currently enrolled in education; and HIV incidence as a primary endpoint; and multiple secondary outcomes (education enrolment and attendance at endline, changes in reported sexual behaviours between baseline and endline, endline prevalence of *Trichomonas vaginalis* and syphilis, and education attendance and graduation rates).

Many of the study participants achieved Forms 1–6 (73.6%), but only 2.6% attained education above Form 5 or 6. Higher education attainment was associated with a 74% reduced risk of HIV infection and a 75% reduction of risk of Trichomonas vaginalis infection. Potential barriers which may affect students staying in school are some of the sexual behaviour of the population which include age mixing, sexual intercourse with more than 1 partner in the last 12 months and having sexual intercourse with non-regular partners. Therefore, the incentives paid for school attendance and remaining STI free in this study might have an impact in behaviour.

The study has some limitations including first the challenges of tracking and tracing the population under study. Adolescent girls and young women aged 15–22 years are mobile and may be challenging to reach if they are sharing cell phones, and constantly changing numbers. The requirement that 50% of the enrolled population be out-of-school contributes to the risk of losing study participants which could lead to losing power to detect a difference of the intervention. Additionally, the behavioural variables analysed are self-reported and some specific risk factors may be under reported within this cohort and vary from other country level studies. This paper presents a broad description of a cohort of adolescent girls and young women and identifies key socio-demographic and behavioural factors associated with HIV and curable STIs which will be further evaluated at the end of the study as factors associated with HIV incidence.

## Conclusions

Adolescent girls and young women in Eswatini are at high risk for HIV infection. Providing educational incentives offers one mechanism to address various factors that increase the risk of HIV acquisition. Previous studies have shown that education may reduce HIV risk through: increased exposure to information about HIV and prevention methods [18, 19]; improved cognitive skills to make complex decisions [20]; better financial security [21–24], reducing participation in transactional sex for women [11]; and the ability to match with lower risk sex partners [25–27]. This is the first impact evaluation of its size which has recruited 50% out-of-school adolescent girls and young women and expanded educational incentives to include multiple forms of education not just traditional primary and secondary schooling. This study will provide further validation on the ability of educational incentives impact on HIV acquisition and will allow for a better understanding of specific risk factors contributing to HIV acquisition among this high-risk population. This impact evaluation provides additional focus on a vulnerable population, out-of-school adolescent girls and young women, and targets this population even though they are hard to reach.

## Data Availability

The datasets used and/or analyzed during the current study are available from the corresponding author on reasonable request.

## Abbreviations

PLHIV: People living with HIV
UNFPA: United Nations Populations Fund
STI: Sexually transmitted infection
HIV: Human immunodeficiency virus
HSV-2: Herpes simplex virus type 2
AGYW: Adolescent girls and young women
EA: Enumeration area
OVC: Orphans and vulnerable children
NERCHA: National Emergency Response for HIV/AIDS
E: Emalangeni
USD: United States dollar
GBV: Gender-based violence
SWAGAA: Eswatini Action Group Against Abuse
PDA: Personal digital assistants
WIRB: Western Institutional Review Board
SD: Standard deviation
